# Supplementary material for: SLC45A4 is a pain gene encoding a neuronal polyamine transporter
Source: Nature. 2025 Aug 20;646(8084):404–12. doi: 10.1038/s41586-025-09326-y (PMC12507699; doi:10.1038/s41586-025-09326-y)
Supplement: Supplementary file 1 — Supplementary Methods, enhanced pain phenotyping questionnaire details and Supplementary Tables 1 and 4–9. [file 41586_2025_9326_MOESM1_ESM.pdf]

nature portfolio
https://doi.org/10.1038/s41586-025-09326-y

Supplementary information

SLC45A4 is a pain gene encoding a neuronal polyamine transporter

In the format provided by the authors and unedited

Supplementary information

Supplementary Methods

Neuromuscular junction analysis macro

BETTER AREA MACRO:

```
//macro altered by Ulrike Schulze. This macro uses a manually selected image part, subtracts a background,enhances contrast,

// and uses Otsu to make a mask. Output is a summary file and results file with area (in um) and grey value statistics.


roiManager("reset");

run("Add to Manager");

title = getTitle();

path = getDirectory("image");

run("Duplicate...", "title=image-2");

run("8-bit");


//makes rectangle and crops the image

setTool("rectangle");

makeRectangle (524, 148, 266, 308);

waitForUser( "Pause","select rectangle ROI and press ok");

run("Crop");


//subtracts background

run("Duplicate...", "title=orig");

selectWindow("image-2");

run("Subtract Background...", "rolling=60");
```

```
//enhances contrast
run("Enhance Contrast...", "saturated=0.001");

//thresholds background subtracted image by using "Intermodes"
run("Duplicate...", "title=area");
run("Threshold...");
waitForUser( "press ok when ready");
run("Convert to Mask");
run("Despeckle");

// process mask, smooth
selectWindow("area");
run("Open");
run("Median...", "radius=4"); //median smoothes edges a bit
run("Convert to Mask");
rename("area");

waitForUser("use paintbrush to remove extraneous objects");

//measure
run("Set Measurements...", "area mean min perimeter integrated display redirect=[orig] decimal=3");
run("Analyze Particles...", "size=1.25-Infinity circularity=0.00- 1.00 show=Masks display summarize add");
//outlines, bare outlines, ellipses will always include the holes in the structure=> don't use

close("image-2");
close("Mask of area");
```

```
dir2 = getDirectory("Output");

selectWindow("Results");

saveAs("Results", dir2+"Results.xls");

selectWindow("Summary");

saveAs("Text", dir2+"Summary.txt");

selectWindow("area");

run("Flatten");

rename("area_image.tif");

saveAs("Tiff", dir2+"area_image.tif");

run("Close");

selectWindow("orig");

saveAs("Tiff", dir2+"orig.tif");
```

Enhanced Pain Phenotyping Questionnaire details

UK Biobank comprises 501,518 volunteers recruited between 2006 and 2010 from across Great Britain. During the initial self-assessment of the recruited participants a brief set of questions screening for the presence, intensity and localisation of chronic pain were completed. This set did not include validated questionnaires and did not differentiate between neuropathic pain form non-neuropathic pain.

In 2019 a detailed "Experience of Pain" self-assessment questionnaire was administered to all UKB participants that had valid email addresses as part of the electronic follow-up (335,587). Developed with input from leading experts and harmonised with large national and international cohorts (Hébert et al., 2023) this questionnaire uses validated questionnaires to ensure a detailed and accurate assessment of pain. By gathering specific information on pain location, intensity, quality, and related conditions like depression and fatigue, the questionnaire provides a richer understanding of chronic pain (Baskozos et al., 2023).

It covers several key areas through different sections, including:

- Screening for medical conditions that frequently lead to the development of chronic pain
- Screening for chronic pain (> 3months) and detailed location and intensity of pain for each one of the listed body sites. Identification and intensity for the most bothersome pain
- Self-completed screening tool for Neuropathic Pain (DN4 (Bouhassira et al., 2005)) related to the most bothersome pain
- Self-reported detailed pain inventory (BPI (Cleeland & Ryan, 1994))
- Self-completed screening tool for Neuropathy (MNSI (Feldman et al., 1994)) if participants reported cancer pain, diabetes, or nerve damage other than diabetic neuropathy
- Health related quality of life (EQ-5D-5L (Herdman et al., 2011))
- Self-completed psychosocial questionnaires about depression (PHQ (Kroenke et al., 2001)) and fatigue (FSS (Krupp et al., 1989))

Baskozos, G., Hébert, H. L., Pascal, M. M. V., Themistocleous, A. C., Macfarlane, G. J., Wynick, D., Bennett, D. L. H., & Smith, B. H. (2023). Epidemiology of neuropathic pain: An analysis of prevalence and associated factors in UK Biobank. Pain Reports, 8(2). https://doi.org/10.1097/PR9.0000000000001066

Bouhassira, D., Attal, N., Alchaar, H., Boureau, F., Brochet, B., Bruxelle, J., Cunin, G., Fermanian, J., Ginies, P., Grun-Overdyking, A., Jafari-Schluep, H., Lantéri-Minet, M., Laurent, B., Mick, G., Serrie, A., Valade, D., & Vicaut, E. (2005). Comparison of pain syndromes associated with nervous or somatic lesions and development of a new neuropathic pain diagnostic questionnaire (DN4). Pain, 114(1–2), 29–36. https://doi.org/10.1016/J.PAIN.2004.12.010

Cleeland, C. S., & Ryan, K. M. (1994). Pain assessment: global use of the Brief Pain Inventory. Annals of the Academy of Medicine, Singapore, 23(2), 129–138. https://europepmc.org/article/med/8080219

Feldman, E. L., Stevens, M. J., Thomas, P. K., Brown, M. B., Canal, N., & Greene, D. A. (1994). A Practical Two-Step Quantitative Clinical and Electrophysiological Assessment for the Diagnosis and Staging of Diabetic Neuropathy. Diabetes Care, 17(11), 1281–1289. https://doi.org/10.2337/DIACARE.17.11.1281

Hébert, H. L., Pascal, M. M. V, Smith, B. H., Wynick, D., & Bennett, D. L. H. (2023). Big data, big consortia, and pain: UK Biobank, PAINSTORM, and DOLORisk. PAIN Reports, 8(5), e1086. https://doi.org/10.1097/PR9.0000000000001086

Herdman, M., Gudex, C., Lloyd, A., Janssen, M., Kind, P., Parkin, D., Bonsel, G., & Badia, X. (2011). Development and preliminary testing of the new five-level version of EQ-5D (EQ-5D-5L). Quality of Life Research, 20(10), 1727–1736. https://doi.org/10.1007/S11136-011-9903-X/TABLES/5

Kroenke, K., Spitzer, R. L., & Williams, J. B. W. (2001). The PHQ-9. Journal of General Internal Medicine, 16(9), 606–613. https://doi.org/10.1046/J.1525-1497.2001.016009606.X

Krupp, L. B., Larocca, N. G., Muir Nash, J., & Steinberg, A. D. (1989). The Fatigue Severity Scale: Application to Patients With Multiple Sclerosis and Systemic Lupus Erythematosus. Archives of Neurology, 46(10), 1121–1123. https://doi.org/10.1001/ARCHNEUR.1989.00520460115022

	Data source	rsID	Genomic coordinates (GRCh38)	effect allele (non effect allele)	EAF	Beta	SE	P-value	Position	eQTL	Gene prioritisation (L2G)	CADD
The leading variants	UKB (EU)	rs3905668	3:136212744	G (A)	0.27	0.02	0.004	1.22×10^{-8}	intergenic	*MSL2, PCCB, PPP2R3A*	*MSL2* (0.28)	4.25
		rs10625280	8:141213072	TAGAC (T)	0.59	0.018	0.003	3.37×10^{-8}	intronic	*SLC45A4, DENND3*	*SLC45A4* (0.49)	1.79
		rs3739238	8:141212346	C (T)	0.59	0.018	0.003	3.63×10^{-8}	exonic	*SLC45A4, DENND3*	*SLC45A4* (0.86)	13.84
SLC45A4 variants replications												
	MVP pain intensity (EU)	rs10625280	8:141213072	TAGAC(T)	0.42	0.026	0.005	1.21×10^{-8}				
		rs3739238	8:141212346	C(T)	0.41	0.026	0.005	7.24×10^{-9}				
	MVP pain intensity (EU male)	rs10625280	8:141213072	TAGAC(T)	0.42	0.027	0.005	8.18×10^{-9}				
		rs3739238	8:141212346	C(T)	0.41	0.027	0.005	4.57×10^{-9}				
	FinnGen pain	rs10625280	8:141213072	TAGAC(T)	0.61	0.021	0.005	1.84×10^{-5}				
	FinnGen pain	rs3739238	8:141212346	C(T)	0.61	0.021	0.005	1.99×10^{-5}				

Supplementary table 1. Lead variants associated with pain intensity (most bothersome) and replications.

Genome-wide significant top variants from UKB pain intensity (most bothersome) GWAS (European ancestry). The lead variant (rs10625280) and missense variant (rs3739238) of *SLC45A4*. Replications were completed in 1. the MVP pain intensity GWAS (European ancestry), 2. European male pain intensity GWAS, and 3. FinnGen pain GWAS. EAF, effect allele frequencies; SE, standard error; eQTL, expression quantitative trait loci; L2G, locus-to-gene pipeline score; CADD, combined annotation dependent depletion.

	*Hs*SLC45A4 LMNG:CHS (EMDB-51377) (PDB 9GIU)	*Hs*SLC45A4 Nanodiscs (EMDB-51365) (PDB 9GHZ)
**Data collection and processing**		
Magnification	165,000x	105,000x
Voltage (kV)	300	300
Electron exposure (e–/Å^2)	57.6	39.71
Defocus range (μm)	-0.6 to -2.5	-0.75 to -2.00
Pixel size (Å)	0.732	0.832
Symmetry imposed	C1	C1
Initial particle images (no.)	10,040,401	20,196,090
Final particle images (no.)	700,436	227,752
Map resolution (Å)	2.83	3.25
FSC threshold	0.143	0.143
Map resolution range (Å)	2.43-16.59	2.91-8.39
**Refinement**		
Initial model used (PDB code)		9GIU
Model resolution (Å)	2.83	3.25
FSC threshold	0.143	0.143
Model resolution range (Å)	2.43-16.59	2.91-8.39
Map sharpening *B* factor (Å^2)		-163
Model composition		
Non-hydrogen atoms	4128	4307
Protein residues	493	500
Ligands	8	14
Waters	17	12
B factors (Å^2)		
Protein	48.06	63.25
Ligand	63.52	76.71
Waters	45.86	56.11
R.m.s. deviations		
Bond lengths (Å)	0.003	0.003
Bond angles (°)	0.534	0.538
Validation		
MolProbity score	1.38	1.52
Clashscore	6.94	7.33
Poor rotamers (%)	0.00	0.24
Ramachandran plot		
Favored (%)	98.36	97.37
Allowed (%)	1.64	2.63
Disallowed (%)	0.00	0.00

Supplementary table 4. CryoEM data collection, refinement and validation statistics.

	WT	HET	KO	Total
No. animals	31	51	12	94
% animals	32.9	54.3	12.8	100
No. males	18	24	4	46
No. females	13	27	8	48
Male weight (g)	26.57 ± 0.71 (6)	26.09 ± 0.88 (7)	25.43 ± 1.60 (3)	
Female weight (g)	20.70 ± 1.10 (5)	20.60 ± 1.02 (7)	20.60 ± 1.47 (5)	

Supplementary table 5. Transgenic mouse viability. Data collected from Het x Het breeding. Fewer KO mice are born than expected. Weights were collected at 8.5-10wks of age. Mean ± SD (n).

	Small sensory neurons					
IB4 binding	IB4-positive			IB4-negative		
Genotype (n)	WT (9)	KO (10)	Test	WT (11)	KO (13)	Test
Capacitance (pF)	16.28 ±1.77	17.40 ±1.75	t-test P = 0.65	17.27 ±1.60	17.27 ±1.41	t-test P = 0.99
RMP (mV)	-49.05 ±2.42	-49.74 ±2.25	MW test P = 0.66	-53.77 ±2.51	-52.23 ±1.24	MW test P = 0.23
Input resistance (mΩ)	320.7 ±46.53	361.1 ±39.64	MW test P = 0.30	394.4 ±33.65	368.0 ±45.67	t-test P = 0.66
Rheobase (pA)	78.97 ±11.45	72.51 ±8.32	t-test P = 0.65	79.57 ±10.85	119.9 ±25.54	MW test P = 0.39

Supplementary table 6. Passive and active membrane properties of WT and *Slc45a4* KO small sensory neurons. Data presented as mean ± s.e.m.

Genotyping Primer	Primer	Sequence	Length
Common Primer	Fwd	TAAAATGGGGAGTCTTGCTGATCTT	25
Mutant Primer	Rev	TAAAGGCCTGGAAGGTGTGGATT	23
Wild type Primer	Rev	GACCACTATGTTGCTGGTACTGA	23
**qPCR Primer**	**Primer**	**Sequence**	**Length**
Slc45a4 (exons 4-5)	Fwd	TTCGTGCCTACCTGCTGGATGT	22
	Rev	GCGTCTGGAACCAGTCACCTA	21
Slc45a4 (exons 5-6)	Fwd	CTCACTTGGTTCTCCGTCATC	21
	Rev	ATCTTCACGCCAGCATTGTA	20
mu HPRT	Fwd	GTCCTGTGGCCATCTGCCTAG	21
	Rev	TGGGGACGCAGCAACTGACA	20
mu Beta actin	Fwd	CATTGCTGACAGGATGCAGAAGG	23
	Rev	TGCTGGAAGGTGGACAGTGAGG	22
mu GAPDH	Fwd	TGTGTCCGTCGTGGATCTGA	20
	Rev	TTGCTGTTGAAGTCGCAGGAG	21

Supplementary table 7. Primers used for genotyping and qPCR

Primary Antibody	Source	Identifier
Rb NeuN (1:500) [EPR12763 monoclonal]	Abcam	ab177487
Ms βIII-Tubulin (1:500) [# TuJ-1 monoclonal]	R&D Systems	MAB1195
βIII-Tubulin-FITC conjugated (1:500) [EP1569Y monoclonal]	Abcam	ab224978
Sh CGRP (1:500) [polyclonal]	Enzo	BML-CA1137
Rb CGRP (1:500) [polyclonal]	BMA Biomedicals	T-4032
IB4, streptavidin conjugated (1:100)	Sigma	L2140
Ch NF200 (1:5000) [polyclonal]	Abcam	ab4680
Sh TH (1:500) [polyclonal]	Millipore	AB1542
Rb TH (1:500) [polyclonal]	Millipore	AB152
Ms anti-FLAG (WB: 1:5000, ICC 1:200) [M2, monoclonal]	Merck	F1804
Ms anti-β-actin (1:10000) [AC-74, monoclonal]	Merck	A2228
Rb anti-Na+/K+ ATPase (1:50) [ST0533, monoclonal]	ThermoFisher	MA5-32184
Rb PGP9.5 (1:400) [polyclonal]	Proteintech	14730-1-AP
Rb CGRP (1:400) [polyclonal]	Merck	C8198
**Secondary Antibody**	**Source**	**Identifier**
Rb PcBl (1:250)	Life Technology,	P-10994
Ms PcBl (1:250)	Thermofisher	P31582
Stp PcBl (1:250)	Life Technology	S11222
Sh Alexa 546 (1:500)	Life Technology	A21098
Rb Alexa 488 (1:500)	Life Technology	A11008
Rb Alexa 546 (1:500)	Life Technology	A11010
Stp Alexa 488 (1:500)	Life Technology	S11223
Ch Alexa 488 (1:500)	Abcam	ab150169
Ch Alexa 546 (1:500)	Life Technology	A11040
Stp Alexa 546 (1:500)	Life Technology	S11225
NeuroTrace (1:10)	Life Technology	N21382
Goat anti-Mouse IgG (H+L) AlexaFluor-488 (1:200)	ThermoFisher	(A28175)
Goat anti-Rabbit IgG (H+L) AlexaFluor-647 (1:200)	ThermoFisher	(A-21245)

Supplementary table 8. Antibodies and dilutions used in this study

**Name**	**m/z**
Putrescine (PUT)	126, 167, **211**
Putrescine-D_8 (PUTD8)	128, 174, **219**
Spermidine (SPD)	223, 336, **364**
Spermidine-D_8 (SPDD8)	231, 344, **372**
Spermine (SPM)	376, 489, **517**
Spermine-D_8 (SPMD8)	384, 497, **525**
4-aminobutanoic acid (GABA)	216, 246, **304**

Supplementary table 9: Polyamine standards and their unique mass identifiers
