# Supplementary material for: SLC45A4 is a pain gene encoding a neuronal polyamine transporter
Source: Nature. 2025 Aug 20;646(8084):404–12. doi: 10.1038/s41586-025-09326-y (PMC12507699; doi:10.1038/s41586-025-09326-y)
Supplement: Supplementary file 2 — Reporting Summary [file 41586_2025_9326_MOESM2_ESM.pdf]

nature portfolio

Corresponding author(s): D Bennett & S Newstead

Last updated by author(s): 2025/05/29

Reporting Summary

Nature Portfolio wishes to improve the reproducibility of the work that we publish. This form provides structure for consistency and transparency in reporting. For further information on Nature Portfolio policies, see our Editorial Policies and the Editorial Policy Checklist.

Statistics

For all statistical analyses, confirm that the following items are present in the figure legend, table legend, main text, or Methods section.

n/a	Confirmed	
☐	☒	The exact sample size (*n*) for each experimental group/condition, given as a discrete number and unit of measurement
☐	☒	A statement on whether measurements were taken from distinct samples or whether the same sample was measured repeatedly
☐	☒	The statistical test(s) used AND whether they are one- or two-sided<br>*Only common tests should be described solely by name; describe more complex techniques in the Methods section.*
☐	☒	A description of all covariates tested
☐	☒	A description of any assumptions or corrections, such as tests of normality and adjustment for multiple comparisons
☐	☒	A full description of the statistical parameters including central tendency (e.g. means) or other basic estimates (e.g. regression coefficient) AND variation (e.g. standard deviation) or associated estimates of uncertainty (e.g. confidence intervals)
☐	☒	For null hypothesis testing, the test statistic (e.g. *F*, *t*, *r*) with confidence intervals, effect sizes, degrees of freedom and *P* value noted<br>*Give P values as exact values whenever suitable.*
☒	☐	For Bayesian analysis, information on the choice of priors and Markov chain Monte Carlo settings
☒	☐	For hierarchical and complex designs, identification of the appropriate level for tests and full reporting of outcomes
☐	☒	Estimates of effect sizes (e.g. Cohen's *d*, Pearson's *r*), indicating how they were calculated

Our web collection on statistics for biologists contains articles on many of the points above.

Software and code

Policy information about availability of computer code

Data collection	Thermofisher EPU used to collect data on microscope, ANYMAZE for mouse tracking, pClamp10 for collecting patch-clamp electrophysiology data, LabChart 8 for Skin-nerve data collections, Zeiss Zen Black for confocal imaging. UK Biobank genotyping: the Applied Biosystems UK Bi LEVE Axiom Array by Affymetrix and the Applied Biosystems UK Biobank Axiom Array. Microscopy: ZeissBlack 2012 (V 8 1 0 484)
Data analysis	SIMPLE3.0 - available on https://github.com/hael/SIMPLE<br>cryoSPARC 3.3.1 (https://cryosparc.com/)<br>RELION3.l.3 - published and freely available<br>PHENIX v1.20.1-4487 - published and freely available<br>Coot v0.9.8.1 EL- published and freely available<br>ISOLDE v 1.6- published and freely available<br>MolProbity v4.4 - published and freely available<br>PyMol v2.6.2- published and freely available<br>ChimeraX 1.5rc202211091945 - published and freely available<br>Graph Pad PRISM 10 - commercially available<br>ImageJ/Fuiji - 1.54g, freely available<br>ZeissBlue (v3.9) - commercially available<br>Cellpose 2.0 - freely avalible<br>Clampfit 10 software - commercially availible<br>KING software (version 2.3.2),<br>PLINK (version l.90b6.21,https://www.cog-genomics.org/plink/l.9/),<br>PLINK2 (version 2.00a5, https://www.cog-genomics.org/plink/2.0/)

REGENIE (version 3.4.1),
R version4 .3.3,
Shimadzu GCMS solution version 2.72 and version 4.50.
Chromsquare software (v2.1.6, Shimadzu)
NIST 11/s, OA_TMS, FA_ME and YUTDI - in house libraries
FUMA v1.5.2 (fuma.ctglab.nl)

For manuscripts utilizing custom algorithms or software that are central to the research but not yet described in published literature, software must be made available to editors and reviewers. We strongly encourage code deposition in a community repository (e.g. GitHub). See the Nature Portfolio guidelines for submitting code & software for further information.

Data

Policy information about availability of data

All manuscripts must include a data availability statement. This statement should provide the following information, where applicable:

- Accession codes, unique identifiers, or web links for publicly available datasets
- A description of any restrictions on data availability
- For clinical datasets or third party data, please ensure that the statement adheres to our policy

Data for this study were obtained from the UK Biobank for project "Risk factors for chronic pain," Application ID: 49572. UK Biobank has approval from the North West Multicentre Research Ethics Committee (MREC) as a Research Tissue Bank (RTB) approval, REC reference: 21/NW/0157, IRAS project ID: 299116. This approval means that researchers do not require separate ethical clearance and can operate under the RTB approval. The genetic and phenotypic data generated by UK Biobank analysed during this study are available via the UK Biobank data access process (see http://www.ukbiobank.ac.uk/register-apply/). Detailed information about the genetic data available from UK Biobank is available at http://www.ukbiobank.ac.uk/scientists-3/genetic-data/ and https://biobank.ndph.ox.ac.uk/ukb/refer.cgi?id=807. The genetic and phenotypic data generated by the Million Veteran Program (MVP) during this study are available via the MVP data access process (see https://www.mvp.va.gov/pwa/mvp-data-available-research). The genetic and phenotypic data generated by the FinnGen during this study are available via the Finnen data access process (see https://elomake.helsinki.fi/lomakkeet/124935/lomake.html.) Detailed information about the genetic data available from the FinnGen is available at https://finngen.gitbook.io/documentation/methods/genotype-imputation/genotype-data. Structural data has been deposited in wwPDB, EMD-51377, 9GIU, (https://doi.org/10.2210/pdb9GIU/pdb) and EMD-51365, 9GHZ (https://doi.org/10.2210/pdb9GHZ/pdb). Datasets relating to biochemical assays and studies in the mouse have been made available as source data alongside this manuscript. Any addition data requests may be obtained from the corresponding authors.

Research involving human participants, their data, or biological material

Policy information about studies with human participants or human data. See also policy information about sex, gender (identity/presentation), and sexual orientation and race, ethnicity and racism.

Reporting on sex and gender	Sex was asked in this study and checked by genotype data. We then removed individuals who showed discordances between self-reported sex and genotyped sex.<br>Overall this study contains 132,552 individuals from which (sex, n, freq), Female 74457 0.562,<br>Male 58095 0.438.
Reporting on race, ethnicity, or other socially relevant groupings	We identified five distinct subpopulations within the UK Biobank: African (9,059 samples), Ad Mixed American (605 samples), East Asian (2,572 samples), European (464,586 samples), and South Asian (9,604 samples). Additionally, there were 1,951 samples for which ancestry could not be determined and were thus categorized as missing. For GWAS association analysis we only included European participants.
Population characteristics	The GWAS contains 132,552 individuals of European ancestry from the UK Biobank for a continuous outcome (i.e., pain intensity, the most bothersome chronic pain). The characteristics includes mean age 66.7 +-7.63SD(when completed the questionnaire), sex (Female 74457, Male 58095).
Recruitment	Individuals were used from UK Biobank (project" Risk factors for chronic pain", ID: 49572), all participants gave informed consent.
Ethics oversight	UK Biobank has approval from the North West Multicentre Research Ethics Committee (MREC) as a Research Tissue Bank (RTB) approval, REC reference: 21/NW/0157, IRAS project ID: 299116. This approval means that researchers do not require separate ethical clearance and can operate under the RTB approval.

Note that full information on the approval of the study protocol must also be provided in the manuscript.

Field-specific reporting

Please select the one below that is the best fit for your research. If you are not sure, read the appropriate sections before making your selection.

☒ Life sciences ☐ Behavioural & social sciences ☐ Ecological, evolutionary & environmental sciences

For a reference copy of the document with all sections, see nature.com/documents/nr-reporting-summary-flat.pdf

Life sciences study design

All studies must disclose on these points even when the disclosure is negative.

Sample size	Human sample sizes we chosen based on all data avalible to us i.e. UK Biobank. For mouse work work sample sizes were calculated using power calculations. Using previous data generated in the lab, animal behaviour, primary outcome, evoked behaviour (von Frey mechanical response), unit mouse 7 biological replicates, effect size d =1.786, power 0.8, with an alpha of 0.05, using two tailed t-test. Secondary measures were electrophysiology and anatomy, detemined by additional power calculations and previous data. Sample sizes were not pre-determined for biochemical/transport assays.
Data exclusions	Participants who have withdrawn (153 as of 15/09/2023) were excluded from the analysis. People who self-reported fibromyalgia (fl20009), chronic fatigue syndrome/myalgic-encephalomyelitis (fl20010) or chronic pain all over the body (fl20021) were excluded from the analysis (11,951 with chronic pain and 821 with no chronic pain). Additional quality control measures were applied that further excluded 367 samples where the reported sex did not match the inferred sex from their genetic data, 651 samples with suspected sex chromosome aneuploidy, and 188 samples with more than ten putative third-degree relatives. In total, 1,024 samples were excluded, with some samples falling into multiple exclusion categories. Animal work, thermal gradient test, one heterozygous mouse (female) was excluded from this test due to a camera fault during the 60 min run.
Replication	Structure solved in detergent and lipid nanodisks. Genetic data was replicated in two independent databases/studies, Million Veterans program and FinnGen, all attempts were successful. Animal data was replicated in multiple cohorts of behaviour, cohort replication was successful. Histological data was all replicated in samples collected from multiple animals, each experiment was carried out more than 3 times, in multiple tissues per animal. Electrophysiological data was successfully replicated in multiple independent experiments each on different days in this case biological replication and indpendent replication are equal. In most cases thoughout the manuscript the number of animals is used to demostrate biological replication. Biochemical assays were successfully replicated in biological replicates (e.g. well containing cells) and from at least 3 independent experiments.
Randomization	Randomisation was used prior to animal behaviour. Experimenters would randomly choose an animal cage, and then randomly choose an animal from that cage to be tested. Doing so randomised the order each animal was tested on each testing day. A similar approach was taken to selecting animals for electrophysiology and sample collection for tissue analysis. Samples were blinded so that down stream experiments (i.e. histology and biochemical assays) we blind and randomised, until analysis was complete. Randomisation related to group allocation for cell based assays and human participants was not required or relevant, all cell-based assays were sampled simultaneously and all human particpants that passed QC were included in the study (details of QC in the methods).
Blinding	Investigators were blinded to group allocation during data collection and analysis

Reporting for specific materials, systems and methods

We require information from authors about some types of materials, experimental systems and methods used in many studies. Here, indicate whether each material, system or method listed is relevant to your study. If you are not sure if a list item applies to your research, read the appropriate section before selecting a response.

Materials & experimental systems

n/a	Involved in the study	
☐	☒	Antibodies
☐	☒	Eukaryotic cell lines
☒	☐	Palaeontology and archaeology
☐	☒	Animals and other organisms
☒	☐	Clinical data
☒	☐	Dual use research of concern
☒	☐	Plants

Methods

n/a	Involved in the study	
☒	☐	ChIP-seq
☒	☐	Flow cytometry
☒	☐	MRI-based neuroimaging

Antibodies

Antibodies used

Primary Antibody Source Identifier
Rb NeuN (1:500) Abcam ab177487 [EPR12763 monoclonal]
Ms βIII-Tubulin (1:500) R&D Systems MAB1195 [# TuJ-1 monoclonal]
βIII-Tubulin-FITC conjugated (1:500) Abcam ab224978 [EP1569Y monoclonal]
Sh CGRP (1:500) Enzo BML-CA1137 [polyclonal]
Rb CGRP (1:500) BMA Biomedicals T-4032 [polyclonal]
IB4, streptavidin conjugated (1:100) Sigma L2140
Ch NF200 (1:5000) Abcam ab4680 [polyclonal]
Sh TH (1:500) Millipore AB1542 [polyclonal]
Rb TH (1:500) Millipore AB152 [polyclonal]
Ms anti-FLAG (WB: 1:5000, ICC 1:200) (Merck F1804), [M2, monoclonal]
Ms anti-β-actin (1:10000) (Merck A2228), [AC-74, monoclonal]
Rb anti-Na+/K+ ATPase (1:50) (ThermoFisher MA5-32184), [ST0533, monoclonal]
Rb PGP9.5 (1:400) Proteintech 14730-1-AP [polyclonal]

Rb CGRP (1:400) Merck C8198 [polyclonal]
Secondary Antibody Source Identifier
Rb PcBI (1:250) Life Technology, P-10994
Ms PcBI (1:250) Thermofisher P31582
Stp PcBI (1:250) Life Technology S11222
Sh Alexa 546 (1:500) Life Technology A21098
Rb Alexa 488 (1:500) Life Technology A11008
Rb Alexa 546 (1:500) Life Technology A11010
Stp Alexa 488 (1:500) Life Technology S11223
Ch Alexa 488 (1:500) Abeam ab150169
Ch Alexa 546 (1:500) Life Technology A11040
Stp Alexa 546 (1:500) Life Technology S11225
NeuroTrace (1:10) Life Technology N21382
Goat anti-Mouse lgG (H+L) AlexaFluor-488 (1:200) (ThermoFisher A28175)
Goat anti-Rabbit lgG (H+L) AlexaFluor-647 (1:200)(ThermoFisher A-21245)

Validation

All validation data was taken from the associated manufacturers websites

Rb NeuN (1:500) Abcam ab177487 [EPR12763 monoclonal]
Anti-NeuN antibody [EPR12763] - Neuronal Marker (ab177487) was developed by Abcam using patented rabbit monoclonal antibody technology and is validated for use in Flow Cyt (Intra), ICC/IF, IHC (PFA fixed), IHC-Fr, IHC-P, WB, mIHC in cat, common marmoset, dog, human, mouse, rat, sheep, zebrafish samples. Abcam's high quality manufacturing and validation processes ensure NeuN antigen antibody (ab177487) has high sensitivity and specificity alongside high lot-to-lot consistency and reproducibility.

Ms βIII-Tubulin (1:500) R&D Systems MAB1195 [# TuJ-1 monoclonal]
Detects mammalian and chicken neuron-specific beta -III tubulin but not other beta -tubulin isotypes in Western blots.

βIII-Tubulin-FITC conjugated (1:500) Abcam ab224978 [EP1569Y monoclonal]
Rabbit Recombinant Monoclonal Beta-3-tubulin antibody - conjugated to FITC. Suitable for ICC/IF, Flow Cyt (Intra) and reacts with Human samples. KO validated.

Sh CGRP (1:500) Enzo BML-CA1137 [polyclonal]
Immunogen synthetic peptide corresponding to a portion of rat α-calcitonin gene-related peptide (CGRP). Likely to react with other mammalien species (based on homology, not tested). Test tissues: Rat thoracolumbar spinal cord.

Rb CGRP (1:500) BMA Biomedicals T-4032 [polyclonal]
This antibody has been tested and validated in ELISA against α-CGRP. Other applications like immunohistochemistry (IHC), FACS or Western Blot may work as well. Optimal dilutions should be determined by the end user.

IB4, streptavidin conjugated (1:100) Sigma L2140
Agglutination activity is expressed in µg/mL and is determined from serial dilutions of a 1 mg/mL solution using phosphate buffered saline, pH 6.8, containing, for each lectin, calcium, magnesium, and manganese at different concentrations. This activity is the lowest concentration to agglutinate a 2% suspension of appropriate erythrocytes after 1 hr incubation at 25 °C.

Ch NF200 (1:5000) Abcam ab4680 [polyclonal]
Anti-Neurofilament heavy polypeptide antibody (ab4680) is a Chicken Polyclonal antibody and is validated for use in ICC, IHC-FrFl, WB.

Sh TH (1:500) Millipore AB1542 [polyclonal]
Tyrosine Hydroxylase (TH, Tyrosine Monooxygenase). Cross-reacts with all mammalian forms tested to date and some non-mammalian forms. Routinely evaluated by Western Blot on mouse brain lysates. The antibody gives specific labeling of noradrenergic axons in primate cerebral cortex.

Rb TH (1:500) Millipore AB152 [polyclonal]
Tyrosine hydroxylase. By western blot, AB152 selective labels a single band at approximately 62kDa (reduced) corresponding to Tyrosine Hydroxylase. It is expected that the antibody will react with most mammalian and many non-mammalian species. It has been reported that this antibody does not work on paraffin embedded human tissue.

Ms anti-FLAG (Merck F1804), [M2, monoclonal]
The M2 antibody will recognize a FLAG® peptide sequence at the N-terminus, Met-N-terminus, C-terminus, or internal sites of a fusion protein. Binding of the M2 antibody is not dependent on calcium. The monoclonal antibody detects only the target protein band(s) on a Western blot from an E. coli, plant or mammalian crude cell lysate.

Ms anti-β-actin (Merck A2228), [AC-74, monoclonal]
Monoclonal mouse anti-actin antibody was used as a loading control for western blot analysis of immunoprecipitated proteins from rat dorsal root ganglion cocultures. Western blot analysis of MDCK cell lysates were performed using monoclonal anti-actin antibody as a primary antibody.

Rb anti-Na+/K+ ATPase (ThermoFisher MA5-32184), [ST0533, monoclonal]
Western blot was performed and a 110kDa band corresponding to ATP1A1 was observed across cell lines and tissues tested.

Rb PGP9.5 (1:400) Proteintech 14730-1-AP [polyclonal]
Various lysates were subjected to SDS PAGE followed by western blot with 14730-1-AP (UCHL1/PGP9.5 antibody)

Rb CGRP (1:400) Merck C8198 [polyclonal]

Anti-Calcitonin Gene Related Peptide antibody produced in rabbit was used for immunohistochemistry of trigeminal ganglia cell cultures and for immunocytochemistry of mouse lung slices.

Eukaryotic cell lines

Policy information about cell lines and Sex and Gender in Research

Cell line source(s)	Neuro-2A mouse neuroblasts (ATCC CCL-131), Human Embryonic Kidney 293-F (Thermofisher Scientific FreeStyle™ 293-F Cells)
Authentication	Authentication based on supplier informaiton, not authenticated in-house
Mycoplasma contamination	Tested negative for mycoplasma contamination
Commonly misidentified lines (See ICLAC register)	n/a

Animals and other research organisms

Policy information about studies involving animals; ARRIVE guidelines recommended for reporting animal research, and Sex and Gender in Research

Laboratory animals	Wild-type mice: C567Bl/6J. Mutant mice: Slc45a4+/- and Slc45a4-/- on a C567Bl/6J background. All experiments were carried out on adult mice, in particular when the mice were above 10-12 weeks old (when salt/pepper hair colour had normalised, and the experiments could be blinded.)
Wild animals	No wild animals were used
Reporting on sex	Both male and female mice were used, it is reported in the methods how many males and females were used. Histology and metabolomics, samples from males and females. Patch-clamp DRG: 4 (2 males 2 females) WT and 4 (2 males 2 females) SLC45A4 KO mice. Spinal and skin-nerve electrophysiology females (WT, 3 KO 2) males (WT 5 KO 6), Behaviour: A total of 15 (8 male, 7 female) wild type mice, 14 (7 male, 7 female) heterozygous mice, and 7 (3 male, 4 female) homozygous knockout mice were used. No differences were observed based on Sex.
Field-collected samples	No field samples were collected in this study
Ethics oversight	All procedures complied with the UK Animals (Scientific Procedures) Act (1986) and were performed under a UK Home Office Project Licence in accordance with University of Oxford Policy on the Use of Animals in Scientific Research.

Note that full information on the approval of the study protocol must also be provided in the manuscript.

Plants

Seed stocks	n/a
Novel plant genotypes	n/a
Authentication	n/a
